# Low-intensity pulsed ultrasound irradiation attenuates collagen degradation of articular cartilage in early osteoarthritis-like model mice

**DOI:** 10.1186/s40634-023-00672-2

**Published:** 2023-10-23

**Authors:** Yoshitsugu Kojima, Takayuki Watanabe

**Affiliations:** 1 Clinical Pharmacology Research Laboratory, Yokohama University of Pharmacy, 601 Matanocho Totsukaku, Yokohama, Kanagawa, 245-0066 Japan; 2 Planning and Product Development Division, Nippon Sigmax Co., Ltd., 7th Floor, 1-24-1 Nishi-shinjuku, Shinjuku-Ku, Tokyo 160-0023 Japan

**Keywords:** Low-intensity pulsed ultrasound, Early osteoarthritis, Low-dose monoiodoacetic acid, Collagen degradation

## Abstract

**Purpose:**

Osteoarthritis (OA) is a combination of degeneration and destruction of articular cartilage due to mechanical stress, secondary synovitis, and bone remodelling. In recent years, early knee OA, a preliminary stage of structural failure in OA, has attracted attention as a potential target for therapy to prevent the onset of OA. Intra-articular administration of monoiodoacetic acid (MIA) induces OA-like symptoms, and low doses of MIA induce early OA like symptoms. In this experiment, a low-dose of MIA was induced to early OA model mice, which were then irradiated with low-intensity pulsed ultrasound (LIPUS) to examine whether LIPUS improves symptoms of early OA.

**Methods:**

After 4 weeks of LIPUS irradiation, articular cartilage was observed at 1 and 4 weeks. The Osteoarthritis Research Society International (OARSI) scores were calculated using Safranin-O staining results. Cartilage degeneration was detected using Denatured Collagen Detection Reagent (DCDR).

**Results:**

We observed a significant decrease in OARSI scores in the LIPUS irradiated group at week 4. The non-LIPUS group showed widespread areas of double positivity for Type II collagen and DCDR, whereas the LIPUS group showed only a small number of DCDR-positive areas. In addition, macrophage numbers counted in the articular capsule at week 1 showed a significant decrease in the LIPUS irradiated group. Lubricin detection showed that lubricin positive cell number was significantly increased by LIPUS irradiation at week 4.

**Conclusions:**

These results suggest that LIPUS attenuates cartilage degeneration in early OA by relieving inflammation and enhancing the inhibitory effect of lubricin on cartilage degeneration.

## Background

Osteoarthritis (OA) is a combination of degeneration and destruction of articular cartilage due to mechanical stress, secondary synovitis, and bone remodelling [2, 11, 21]. As the most prevalent musculoskeletal disease, OA has one of the fastest growing patient populations worldwide [1, 6]. OA currently affects more than 528 million people globally, suggesting a nearly 10% increase in the last 30 years, since 1990 [6]. OA causes chronic pain and gait disturbance, impairs daily activities, and reduces quality of life [11]. Furthermore, it is a progressive disease that cannot be reversed or stopped after onset. In recent years, early knee OA, a preliminary stage of structural failure in OA, has attracted attention as a potential target for therapy to prevent the onset of OA [20]. While an accepted definition of early-stage OA has not yet been established, it is generally described as a condition in which the symptoms of OA, mainly pain, manifest above a certain level, despite the absence of joint crevice narrowing on plain radiographs [19, 22]. Abnormal findings on magnetic resonance imaging (MRI), such as cartilage defects, meniscus injuries, and osteophytes, are found in patients over 40 years of age, even in those with no specific knee symptoms or history of trauma [7]. Cartilage degeneration, bone marrow lesions, and semilunar deviation on simple MRI were associated with knee pain in a knee joint determined to be KL grade 0 on simple radiographs [36]. It has been suggested that early initiation of treatment may increase the possibility of preventing the onset and progression of OA.

While various OA models have been developed, the monoiodoacetic acid (MIA) administration model is used as one standard OA model. Intra-articular administration of MIA induces inflammation in joints by inhibiting glycation of chondrocytes through inhibition of glyceraldehyde-3-phosphatase dehydrogenase, and induces various degrees of OA progression depending on the dose of MIA administration [28]. The conventional dosage of MIA (1–3 mg) has a strong effect on cartilage destruction [3]. However, a lower dose of MIA can induce cartilage degeneration without bone destruction, which is more similar to the joint degeneration observed in human early-OA. Rats treated with low-dose MIA are reported as animal models of early OA [41].

Low-intensity pulsed ultrasound (LIPUS) is widely used in bone fracture healing [14]. LIPUS irradiation significantly shortens treatment times in patients with bone fractures. Several studies have reported the usefulness of LIPUS for the treatment of bone fractures, delayed union, and non-union [4, 31, 42]. In addition, it affects not only osteoblasts, but also chondrocytes and inflammatory immune cells [25, 33, 44]. Furthermore, LIPUS reportedly provides pain-relieving effects and is, therefore, used to treat patients in advanced stages of OA; improvements in Visual Analogue Scale and Western Ontario and McMaster Universities Arthritis Index scores have been observed [5]. However, these results only demonstrate the analgesic effect of LIPUS in post-onset OA, not its efficacy in inhibiting the onset of OA. We hypothesised that LIPUS can inhibit the progression of early OA. Therefore, in this study, we examined whether LIPUS delays the progression of OA by attenuating cartilage degeneration and increasing lubricin expression in an early OA mouse model.

## Methods

### Ethics statement

All animal experiments conformed to the Guide for the Care and Use of Laboratory Animals of the National Institutes of Health and the ARRIVE guidelines (http://www.nc3rs.org/ARRIVE). All the experiments were approved by the Institutional Committee of Laboratory Animal Experimentation of the authors’ affiliated institutions.

### Animals

Female ICR mice (31–35 g, 13 weeks old) were acclimatised for 1 week before the experiments. The mice were housed under standard laboratory conditions (12-h light/dark cycle, 25 °C). They were provided ad libitum access to water and food containing 0.98% calcium, 0.80% phosphorus, and vitamin D3 (3,169 IU/kg) for pair feeding (Labo MR Stock, Nosan corp., Kanagawa, Japan).

### MIA injection and LIPUS irradiation

Hair from the skin of the right hind limb was removed with depilatory cream (Reckitt Benckiser Japan Ltd., Tokyo, Japan) for LIPUS irradiation. The mice were anaesthetised with isoflurane (Mylan Inc., Pittsburgh, PA, USA) using an animal anaesthesia machine (SFSFB02, DS Pharma Biomedical Co. Ltd., Osaka, Japan) The conditions were as follows: for induction, concentration = 4%, flow rate = 2.8 L/min; for maintenance, concentration = 2%, flow rate = 2.8 L/min. The anaesthetised mice were placed on a heated pad maintained at 35 °C during the operation. The mice were administered with MIA (0.1 mg) by intra-articular injection under anaesthesia [41]. These mice were randomised by body weight into the LIPUS ( −) group (control) and LIPUS ( +) group (experimental). On the day after MIA administration, ultrasound gel was applied to an ultrasound transducer (Nippon Sigmax Co. Ltd., Tokyo, Japan), which was placed over the exposed skin of the right knee. Subsequently, LIPUS treatment was administered 5 days per week for 20 min per day. The ultrasound exposure conditions were as follows: effective transducer area, 6.85 cm^2^; intensity, 30 mW/cm^2^ with a 20% duty cycle; and pulse frequency, 2.0 MHz with a 1 kHz repeat rate. At weeks 1 and 4, mice were euthanised by posterior cervical dislocation, following sufficient isoflurane inhalation, to prepare tissue sections of the knee joint.

### Histological analysis

Mouse whole knee joints were harvested at weeks 1 and 4, fixed with 4% paraformaldehyde (Fujifilm Wako Pure Chemical Corp., Osaka, Japan), demineralised with ethylenediaminetetraacetic acid (Dojindo Laboratories, Kumamoto, Japan), and embedded in paraffin to prepare 5-μm-thick sections of frontal plane tissue. Histological sections were visualised under a fluorescence microscope (BZ-9000; Keyence, Osaka, Japan).

#### Safranin O staining

Safranin O staining results were used to calculate the osteoarthritis research society international (OARSI) scores of all four quadrants of the joint: medial femoral condyle (MFC), medial tibial plateau (MTP), lateral femoral condyle (LFC), and lateral tibial plateau (LTP) [10]. A score of 0 represents normal cartilage, 0.5 = loss of PG with an intact surface, 1 = superficial fibrillation without loss of cartilage, 2 = vertical clefts and loss of surface lamina (any % or joint surface area).

#### Immunofluorescence staining

To detect degenerated collagen on the articular cartilage, the tissue sections were treated with Denatured Collagen Detection Reagent (DCDR; Funakoshi Co., Ltd., Tokyo, Japan) [39, 40]. They were then treated with anti-type II collagen antibody (Santa Cruz Biotechnology, Inc., Santa Cruz, CA, USA). The tissue sections were treated with F4/80 antibody (Bio-Rad Laboratories Inc., Hercules, CA, USA) [8] and anti-IL-6 antibody (Cell Signaling Technology, Danvers, MA, USA). The tissue sections were treated with anti-Aggrecan antibody or anti- Matrix metalloproteinase 13 (MMP-13) antibodies (Proteintech Group Inc., IL, USA). The tissue sections were treated with anti-type II collagen antibody and anti-lubricin antibody (Novus Biologicals, LLC., Central CO, CO, USA). The immunofluorescence-positive cells in the articular capsule were counted using the ImageJ software [29, 34].

### Statistical analysis

The primary goal of this study was to observe the histological changes of MIA early-OA model, with and without LIPUS irradiation. Our sample size was determined by referring to a previous study that examined OARSI histological score in a mouse MIA OA model [41]. Calculations were performed according to this difference with 90% statistical power and α level of 0.05, and it was determined that at least 4 mice were needed. Parametric data are shown as mean ± standard error of the mean. P-values of < 0.05 were considered statistically significant. The significance of differences between the two groups was determined using an independent sample T test. Data were processed using the Ekuseru–Toukei 2012 software (Social Survey Research Information Co. Ltd., Tokyo, Japan).

## Results

**Fig. 1:**
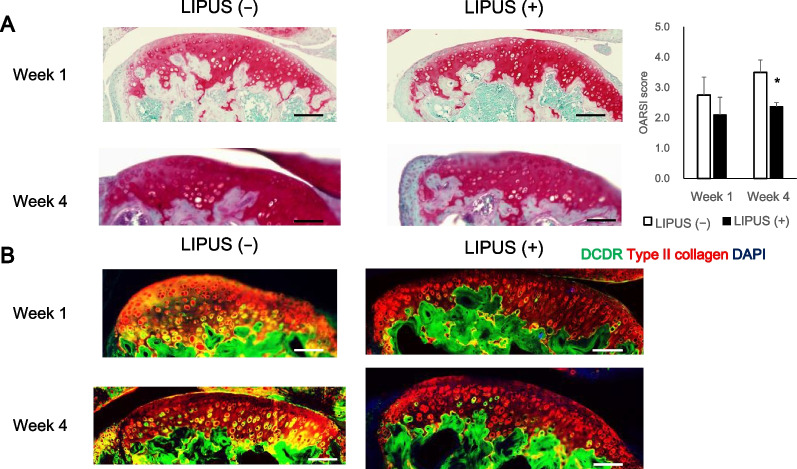
LIPUS attenuates cartilage degeneration in early osteoarthritis-like model mice. **A** Safranin O-stained histology sections of articular cartilage after weeks 1 and 4. The results were used to calculate the osteoarthritis research society international (OARSI) scores of all four quadrants of the joint: medial femoral condyle (MFC), medial tibial plateau (MTP), lateral femoral condyle (LFC), and lateral tibial plateau (LTP). Values are expressed as mean ± standard error of the mean (SEM), *n* = 4; * = *p* < 0.05. (Scale bar: 100 μm). **B** Collagen degeneration on the articular cartilage was detected with Denatured Collagen Detection Reagent (green). Type II collagen was immune-stained (red). (Scale bar: 100 μm)

We compared cartilage degeneration in the LIPUS ( −) and LIPUS ( +) groups for Safranin O staining (Fig. 1A). At week 1, there was nearly no loss of safranin and no significant difference between groups; however, at week 4, a significant increase of OARSI score was observed in the LIPUS ( −) group. In addition, collagen denaturation of cartilage was detected by DCDR (Fig. 1B). DCDR is a reagent that binds specifically to collagen fibres that have not formed a triple helix in denaturation [39]. At both weeks 1 and 4, the LIPUS ( −) group showed widespread areas of double positivity for type II collagen and DCDR, whereas the LIPUS ( +) group showed few DCDR-positive areas. These results suggest that LIPUS irradiation inhibits low-dose MIA-induced cartilage degeneration.

**Fig. 2:**
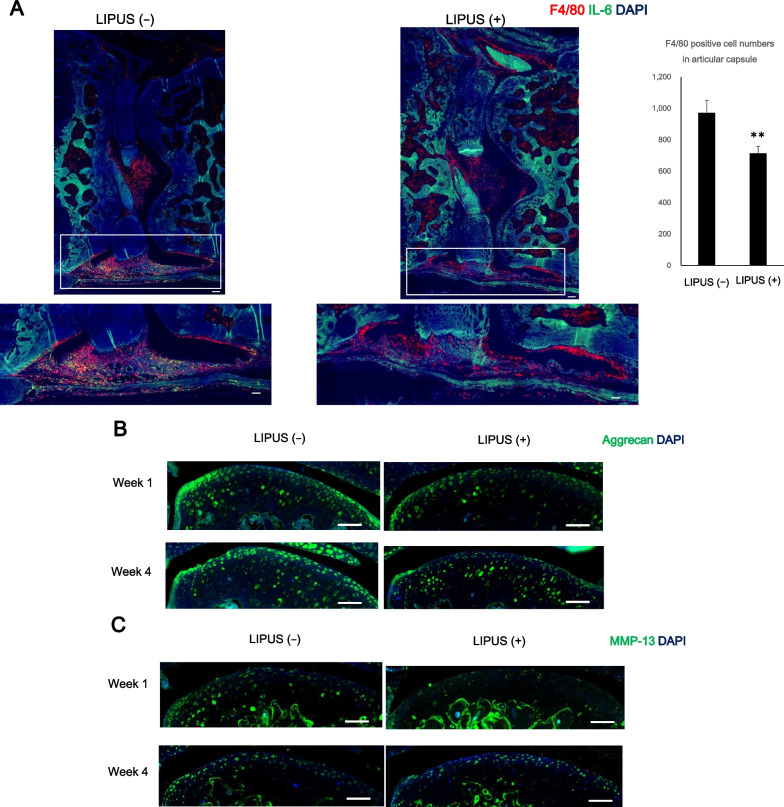
LIPUS attenuates infiltration of F4/80 positive macrophage in early osteoarthritis-like model mice. **A** Immunofluorescent staining of mouse knee arthrodesis using F4/80 antibody (red) and anti-IL-6 antibody (green). F4/80 positive cells were counted. Values are expressed as mean ± standard error of the mean (SEM), *n* = 4; ** = *p* < 0.01. (Scale bar: 100 μm). **B** Immunofluorescent staining of mouse articular cartilage at weeks 1 and 4, using anti-Aggrecan antibody (Green). (Scale bar: 100 μm). **C** Immunofluorescent staining of mouse articular cartilage at weeks 1 and 4, using anti-MMP-13 antibody (Green). (Scale bar: 100 μm)

To verify whether LIPUS attenuates cartilage degeneration by suppressing inflammation, the number of F4/80-positive cells in the articular capsule was measured (Fig. 2A). Infiltration of inflammatory cells was observed by staining of the F4/80 molecule, one of the markers of mouse macrophage tissue [8]. In week 1, there was a significant decrease in the number of F4/80 positive cells in the LIPUS ( +) group compared with the LIPUS ( −) group. Aggrecan positive cells and MMP-13 positive cells were also observed (Fig. 2B and C). No significant difference was observed in the Aggrecan-positive cells with or without LIPUS irradiation; however, the MMP-13-positive cells in the LIPUS (–) group were found to be particularly abundant in the middle zone in week 1.

**Fig. 3:**
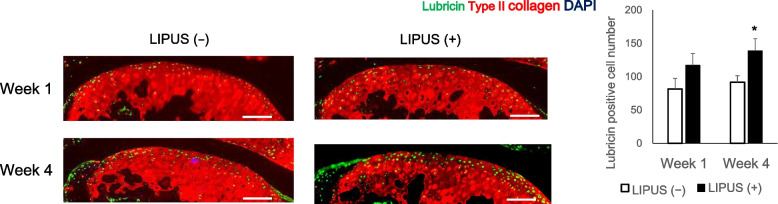
Immunofluorescent staining against lubricin (green) and type II collagen (red) in the articular cartilage sections. Lubricin-positive cells were counted. Values are expressed as mean ± standard error of the mean (SEM), *n* = 4; * = *p* < 0.05. (Scale bar: 100 μm)

Mechanical stress on the surface layer of cartilage, such as shear stress and running, induces lubricin expression [26]. Therefore, we examined whether LIPUS irradiation increases lubricin expression. The lubricin positive cell numbers in the superficial layer of cartilage were increased in the LIPUS ( +) group (Fig. 3). These findings suggest that LIPUS suppresses inflammation and cartilage degeneration in early OA.

## Discussion

Matrix metalloproteinases (MMPs) and a disintegrin and metalloproteinase with thrombospondin motifs (ADAMTSs) degrade type II collagen and aggrecan, and exacerbate OA; their expression is promoted by inflammation [43]. In addition, these fragments of cartilage component proteins stimulate synovial cells and chondrocytes, inducing further inflammation and progression of OA [12, 38]. Therefore, suppression of inflammation within OA joints is considered important for maintaining joint function [38]. In this study, LIPUS irradiation reduced macrophage infiltration into the joint capsule in the acute inflammation 1 week after MIA administration, suggesting an inhibitory effect on inflammation. LIPUS promotes the secretion of extracellular vesicles and the suppression of inflammation [18].

Lubricin is a glycoprotein secreted into synovial fluid by superficial zone chondrocytes and synoviocytes [17, 32]. It reduces and protects the frictional resistance of the cartilage surface and plays an important role in maintaining cartilage homeostasis. Since TRPV2, a mechano-sensor in the superficial zone of articular cartilage, induces lubricin via cAMP response element binding protein (CREB), we postulated that LIPUS also activates this pathway [24]. Our study found that the number of lubricin-positive cells was increased in the LIPUS-irradiated cartilage surfaces. Furthermore, LIPUS suppresses MMP13 production from chondrocytes [15, 23, 27, 35]. These results suggest that LIPUS induces lubricin expression to provide further protection to cartilage integrity.

This study had some limitations. We showed that LIPUS irradiation suppressed inflammation and cartilage degeneration in early OA mice. However, deviation of the medial meniscus is frequently observed in patients with early OA [13]. Because the study was conducted only on MIA-induced OA mice, validation using surgical OA model mice, such as ligament-tearing OA models, is needed to examine the effects in human OA patients [16]. Next, although a histological evaluation was performed in this study, RNA should also be extracted from cartilage and analysed by reverse transcription-quantitative polymerase chain reaction (RT-qPCR). This is because quantitative expression analysis using RT-qPCR has become an indispensable research method in the field of orthopaedics to elucidate the molecular mechanisms of pathogenesis and disease progression.

The efficiency of early case detection and diagnosis in primary care enables health care providers to proactively and significantly reduce the burden of disease through appropriate management, such as by addressing certain lifestyle-related risk factors for disease progression [37]. One of the reasons for focusing on early-stage OA is to prevent the progression of OA by starting treatment early [9]. Although moderate exercise can increase lubricin expression [30], LIPUS irradiation may improve symptoms in patients having difficulty with physical therapy. Intervention in early OA at the onset of pain using LIPUS, a non-invasive technique, may reduce the incidence of OA. By investigating the ultrasound irradiation conditions optimised for lubricin induction and inflammation suppression, LIPUS may serve as a potential candidate for the treatment of early OA.

## Data Availability

The datasets used and analysed during the current study are available from the corresponding author on reasonable request.

## Abbreviations

OA: Osteoarthritis
MIA: Monoiodoacetic acid
LIPUS: Low-intensity pulsed ultrasound
OARSI: Osteoarthritis research society international
DCDR: Denatured Collagen Detection Reagent
MMP: Matrix metalloproteinase
ADAMTSs: A disintegrin and metalloproteinase with thrombospondin motifs
CREB: CAMP response element binding protein
